# Enhancing Doctor-Patient Shared Decision-Making: Design of a Novel Collaborative Decision Description Language

**DOI:** 10.2196/55341

**Published:** 2025-03-04

**Authors:** XiaoRui Guo, Liang Xiao, Xinyu Liu, Jianxia Chen, Zefang Tong, Ziji Liu

**Affiliations:** 1 School of Computer Science Hubei University of Technology Wuhan China

**Keywords:** shared decision-making, speech acts, agent, argumentation, interaction protocol

## Abstract

**Background:**

Effective shared decision-making between patients and physicians is crucial for enhancing health care quality and reducing medical errors. The literature shows that the absence of effective methods to facilitate shared decision-making can result in poor patient engagement and unfavorable decision outcomes.

**Objective:**

In this paper, we propose a Collaborative Decision Description Language (CoDeL) to model shared decision-making between patients and physicians, offering a theoretical foundation for studying various shared decision scenarios.

**Methods:**

CoDeL is based on an extension of the interaction protocol language of Lightweight Social Calculus. The language utilizes speech acts to represent the attitudes of shared decision-makers toward decision propositions, as well as their semantic relationships within dialogues. It supports interactive argumentation among decision makers by embedding clinical evidence into each segment of decision protocols. Furthermore, CoDeL enables personalized decision-making, allowing for the demonstration of characteristics such as persistence, critical thinking, and openness.

**Results:**

The feasibility of the approach is demonstrated through a case study of shared decision-making in the disease domain of atrial fibrillation. Our experimental results show that integrating the proposed language with GPT can further enhance its capabilities in interactive decision-making, improving interpretability.

**Conclusions:**

The proposed novel CoDeL can enhance doctor-patient shared decision-making in a rational, personalized, and interpretable manner.

## Introduction

Shared decision-making (SDM) is a collaborative process between health care providers and patients, designed to incorporate the patient’s preferences, values, and the best available evidence to discuss and agree on the most suitable care options [1]. Numerous studies have demonstrated the benefits of SDM, including improved patient engagement in decision-making, enhanced quality of care, and reduced medical costs [2]. In recent years, SDM has emerged as the pinnacle of clinical decision-making, particularly in evidence-based and patient-centered health care settings [3-5].

However, opportunities for active patient participation in medical decision-making remain limited [6], and SDM is predominantly applied in specific health care scenarios [7]. Furthermore, considerable disagreement exists regarding the implementation of SDM, primarily due to the absence of a universally accepted set of steps to guide the process [8,9]. To address these challenges, the Tripartite Dialogue Model was developed as an approach to promote SDM by engaging relevant stakeholders, ensuring continuous updates, and fostering broader participation [10,11]. However, the practical implementation of similar dialogue models remains largely theoretical, with a lack of detailed guidelines for execution. As a result, understanding the process of joint decision-making and addressing the issue of inadequate consultation—often leading to inefficient utilization of medical resources—has proven challenging [12]. Research indicates that reaching an agreement on a practical model would be a crucial step in promoting its adoption. Currently, there is no existing research specifically focused on doctor-patient codecision-making [7].

Communication among agents, akin to the interaction between a doctor and a patient, is a multifaceted and intricate process. Achieving effective communication and seamless information exchange is crucial for agents to collaborate harmoniously and work toward a shared objective. In the field of linguistic analysis, speech acts have gained prominence for capturing expressions of mental states—such as embarrassment, gratitude, or regret—that convey thoughts or ideas and elicit specific behaviors, such as commands, warnings, or requests, to sustain social interaction [13]. Most research on speech acts has primarily focused on areas such as task assignment, knowledge expression, and identifying intrinsic behavioral patterns in language [14,15]. However, the use of speech acts for regulating interaction behavior remains limited. Among the existing studies that leverage language to describe group behavior, a particularly suitable approach is the Lightweight Coordination Calculus (LCC) language. Initially developed within the framework of the Open Knowledge Project [16], LCC was later extended to the Lightweight Social Calculus (LSC) to support the development of social machines [17]. These languages were not specifically designed to address decision problems and therefore require substantial enhancements to be effectively applied in group decision-making scenarios.

Over the years, various interaction mechanisms for group decision-making have been explored. For example, researchers have developed decentralized group decision consensus mechanisms [18] and introduced personalized feedback mechanisms that provide decision makers with varying levels of consensus alongside tailored feedback suggestions [19,20]. However, in the medical domain, there have been relatively few studies focusing on personalized modeling to describe the group decision-making process. The primary reason is that poor decision-making is often influenced by artificial problems, such as cognitive biases, which may stem from social attributes or certain personality traits [21]. To support group decision-making in health care, some researchers have developed clinical decision support systems [22,23]. These systems aim to create human-computer interactive medical platforms that use data or models to assist doctors and patients in clinical decision-making [24]. However, these systems largely depend on third-party knowledge and fail to adequately consider users’ value orientations or preferences.

In medical decision-making, treatment choices are often influenced by physicians’ preferences and experience rather than being solely based on medical necessity or scientific evidence, partly due to the lack of evidence-based guidance on the optimal treatment order [25]. To encourage greater patient participation in SDM, patient decision aids (PtDAs) have been developed. These aids provide standardized information about available options and their associated outcomes, helping patients implicitly evaluate the value of these outcomes [26]. However, many existing PtDAs are text-based, include instructions and training for health professionals, and are not aligned with clinical guidelines [27].

With advancements in machine learning and the availability of powerful hardware supporting real-time speech recognition—such as high-quality text-to-speech capabilities and semantic understanding of natural language—health dialogue systems are becoming increasingly popular among patients, hospitals, and universities [28]. These systems enable patients to acquire knowledge and engage in conversations without the direct involvement of health care professionals. Among these systems, ChatGPT, developed by OpenAI, stands out as a prominent language model with significant potential to contribute to public health [29]. Leveraging its extensive knowledge base, it can address patients’ inquiries, particularly when dealing with uncertain questions. However, it is important to recognize that, despite its vast database, ChatGPT lacks a deep semantic understanding of the messages it processes [30]. This limitation can undermine trust in its responses, especially in the complex field of medicine, where accuracy and reliability are paramount.

Therefore, by integrating the aforementioned aspects, this paper makes the following key contributions:

- We enhance GPT’s explainability by integrating our method with GPT, thereby verifying the feasibility of our approach and enriching our argument.
- We introduce Collaborative Decision Description Language (CoDeL), which includes 4 key elements: (1) a decision-maker interaction process protocol based on LSC; (2) the semantic relationships in the interaction process, defined by combining speech acts; (3) arguments in semantics guided by clinical guidelines; and (4) constraints incorporated for personality modeling. This language provides a more comprehensive description of group decision-making problems and addresses the limitations of LSC.
- We have created some reusable interaction patterns that can be applied in other domains. Specifically, we model the field of atrial fibrillation to validate the feasibility of our method and introduce a novel evaluation approach.

## Methods

### Overview

We first outlined what our LSC inherited and extended from previous studies (the “LSC-Based Decision-Making Protocol” section). Next, we introduced grammatical semantics within our defined CoDeL (the “Syntactic and Semantics of Collaborative Decision Languages [CoDeL]” section) and demonstrated the interactive relationships of speech acts (the “Constraints of Permissible Speech Acts Pairs” section). Based on the communication model for joint decision-making between doctors and patients, we defined 3 distinct decision-making models (the “An Interactive Model of Shared Decision-Making Between Doctors and Patients” section). We then used CoDeL to establish the protocol for these decision-making models (the “Protocols Specification Using Collaborative Decision Description Language [CoDeL]” section). Additionally, we briefly discussed the arguments guided by clinical guidelines. Finally, we incorporated the personality model constraint into the protocol (the “The Personality Modeling Included in the Protocol” section).

### LSC-Based Decision-Making Protocol

LSC is a protocol language designed to regulate social interactions. It provides a framework for creating social machines that build upon existing social networks, such as X (Twitter), while supporting human interactions through formal role binding, protocol enactment, and result interpretation back to human society [31]. By meeting specific conditions, participants can seamlessly integrate into LSC-based systems, with each condition defining a distinct role and corresponding behavior. In practical applications, the LSC protocol comprehensively defines the agents, roles, messages, computations, and information flows involved, ensuring precise control over complex social interactions and guaranteeing system reliability and efficiency. However, the earlier version of LSC lacked a critical decision-making component, as it did not incorporate clinical guidelines or natural language processing capabilities. Specifically, the original LSC lacked mechanisms for processing natural language and did not support dialogues with argumentation structures grounded in logical reasoning.

To address these gaps, we have extended the earlier version of LSC with a new decision-making component that integrates clinical guidelines and natural language processing capabilities. Our primary objective is to develop an integrated framework that facilitates decision-making in medical settings by leveraging evidence-based recommendations and logical reasoning.

Therefore, we propose a CoDeL to provide health care professionals and patients with a more comprehensive decision support system. Specifically, CoDeL contributes in the ways detailed in Textbox 1.

In contrast to the original LSC, our CoDeL is designed to enable more effective and informed decision-making in medical settings. CoDeL, equipped with natural language processing and evidence-based recommendation capabilities, supports the development of comprehensive decision-support systems tailored to the needs of health care professionals and patients. By addressing this critical gap, we aim to improve patient outcomes, enhance health care quality, and empower clinicians with a cutting-edge tool to support clinical decisions.

**Collaborative Decision Description Language (CoDeL) contributions.**

**1. Semantically linked to abstract-level speech acts**

CoDeL formalizes concrete speech acts into an abstract symbolic format, for example, Rebuttal(surgery1, against_argument)), enabling precise linguistic data transmission and ensuring accuracy in communication.

**2. Seamless integration of clinical guidelines**

CoDeL incorporates the latest medical guidelines into the decision-making process, providing clinicians with access to up-to-date, evidence-based recommendations. This integration supports informed decision-making through logical reasoning and argumentation structures, ensuring decisions are grounded in empirical evidence.

**3. Enhanced patient engagement**

CoDeL uses user-friendly natural language formats alongside interactive dialogue mechanisms, enabling patients to participate actively in treatment decision-making. This approach fosters clearer communication, empowering patients to make more informed choices.

### Syntactic and Semantics of Collaborative Decision Languages (CoDeL)

Group decision problems involve analyzing individual opinions and preferences to understand how decisions are made. These problems often require cooperation and negotiation among multiple individuals, taking into account factors such as opinions, preferences, rights, and interests. To address these challenges effectively, we propose CoDeL as a framework for describing and solving group decision-making problems. Specifically, to enhance the scientific validity of CoDeL, we define distinct categories of communication processes within the context of doctor-patient SDM in medical scenarios. This approach facilitates improved communication and negotiation among the involved agents, enabling a more robust decision-making process, as outlined in Textbox 2.

It is crucial to emphasize that within the context of our framework, the term *rebuttal* allows for negotiation, discussion, and the presentation of counterarguments. By contrast, *refuse* denotes a definitive and explicit rejection, devoid of any accompanying explanation or reasoning. The concept of *refuse* carries a more forceful connotation, as it involves an outright dismissal of the proposed action or treatment without providing further justification or elaboration.

In this research, we introduce CoDeL, a framework that not only encompasses the aforementioned interaction behaviors but also incorporates a group decision protocol. This protocol defines the roles of agents, specifies the messages to be exchanged, and outlines the information to be utilized during interactions. The protocol can be executed either sequentially or as a committed-choice option, ensuring a systematic and coordinated decision-making process. Within each action, agents follow the protocol associated with their roles and interact with other agents accordingly. Before taking any action, agents evaluate whether they are authorized to perform the action by verifying compliance with the corresponding behavioral rules.

**Figure 1:**
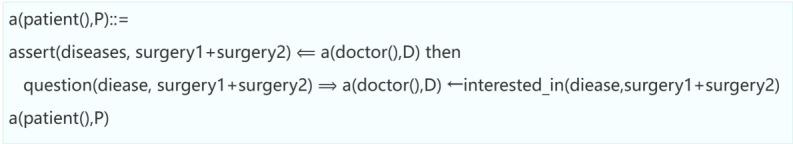
An example of doctor-patient communication is LSC. After receiving a message from the doctor, the patient decides whether to send an inquiry message.

To facilitate a better understanding of the CoDeL syntax, we provide an illustrative example in Figure 1, demonstrating how the framework is applied in practice.

- “a(name(K),N)”: Indicates the agent’s role name, ID, and intrinsic knowledge of existence.
- “Agenti() ⇒ Agentj()” or “Agenti() ⇐ Agentj()”: Indicates that the message is being passed to another agent. A message declaration needs to be specified on the sender side and a rule declaration on the receiver side.
- “then”: Indicates that the clause before it must continue the clause after it.
- “or”: Indicates that there are alternative decision branches available and only 1 should be selected.
- “←()”: Indicates that the constraint satisfaction function must resolve to true in order to execute its associated clause.
- “assert(diseases, surgery1 + surgery2) ⇐ a(doctor(),D)”: The patient receives an assertion message sent by the doctor, which includes the name of the disease and the doctor’s recommended treatment plan.
- “question(disease, surgery1 + surgery2) ⇒ a(doctor(),D)←interested in(disease, surgery1 + surgery2)”: If the patient wishes to acquire more knowledge, they can send a message of inquiry to the doctor and await confirmation from the doctor.

**Decision-making process.**

**1. Assert**

Provide information related to diagnoses, medication instructions, treatment plans, or consultation details.

**2. Accept**

Agree to follow the proposed action plan, treatment plan, or medical advice.

**3. Refuse**

Reject the proposed action plan, treatment plan, or medical advice.

**4. Question**

Seek further clarification due to limited understanding of the information provided in an assertion.

**5. Justify**

Offer honest answers to questions raised and provide reasonable explanations to support assertions made.

**6. Rebuttal**

Disagree with an assertion and present reasons for the opposition.

**7. Persuade**

Attempt to influence the beliefs, attitudes, or behaviors of the opposing party to accept or adopt the assertion made.

**8. Retract**

Withdraw the initial assertion due to the discovery of errors or a change in beliefs based on updated information.

### Constraints of Permissible Speech Acts Pairs

**Figure 2:**
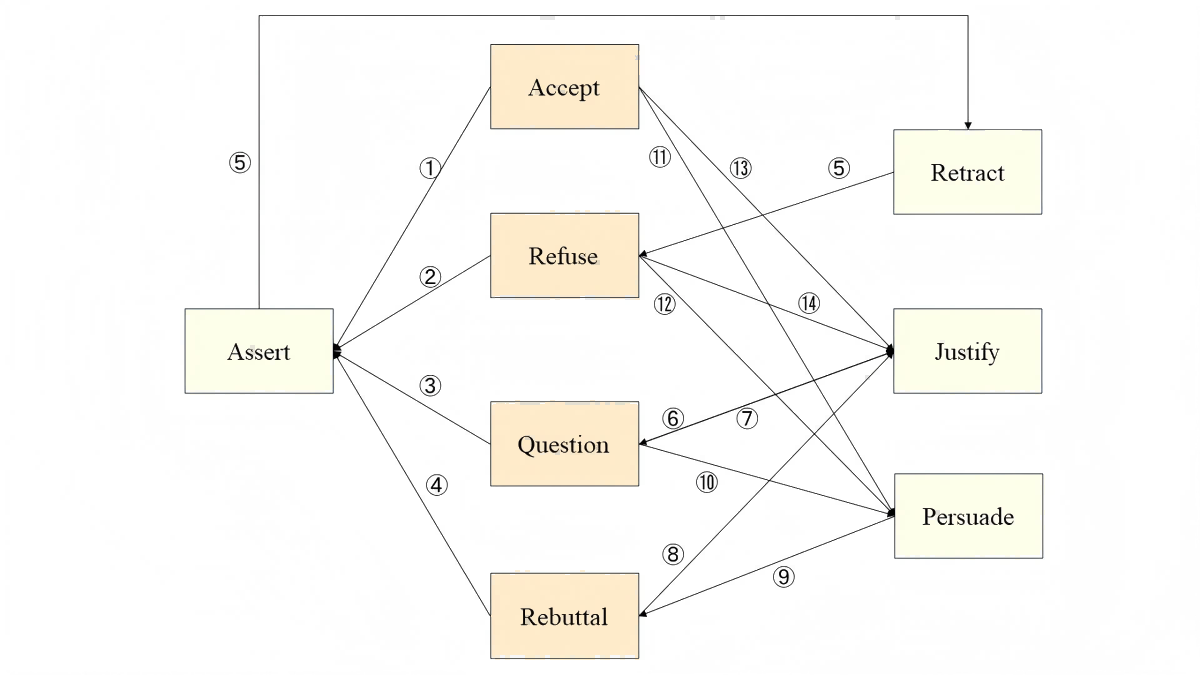
Interaction relationship of speech acts.

Speech acts do not exist in isolation; instead, they intersect and influence each other within the dynamic evolution of dialogue, thereby facilitating communication between agents. Figure 2 illustrates the relationships among speech acts, and Table 1 standardizes and explains the speech act interaction pairs applicable to this study.

For instance, when a doctor makes a diagnostic assertion, the patient’s response can take various forms, including acceptance, rejection, questioning, or refutation (1-4). If the patient questions the initial assertion, the doctor must provide detailed arguments and validate the rationality of the assertion (6). Based on the doctor’s response, the patient may choose whether to continue questioning (7). If the patient rejects the evidence and refutes it (8), the doctor may use persuasive strategies to reinforce their initial position (9), potentially initiating another feedback loop. During this process, the patient may persist with further questions (10), fostering a cycle of in-depth interaction until all concerns are resolved. At the conclusion of the cycle, the patient, influenced by the doctor’s persuasion and verification, ultimately makes 1 of 2 decisions: to accept or reject the doctor’s assertion (11-14). As the conversation progresses and both parties exchange their perspectives, if the patient persists in rejecting a specific assertion, the doctor may decide to actively retract the previous statement after considering the pros and cons (16). The doctor can then determine whether to propose a new assertion based on the available alternatives (5). Notably, certain speech act interaction pairs that violate standard behavioral logic are prohibited during agent interactions. For instance, as described above, an agent sending a *refuse* message demonstrates a strong rejection attitude and will not receive any response message. Consequently, the (*refuse-persuade*) interaction pair is logically invalid. Similarly, the (*assert-justify*) interaction pair, where an agent sends an *assert* message to express its opinion and the receiving agent replies with a *justify* message to provide supporting arguments for the other agent’s opinion, also violates normal behavioral logic.

**Table 1:** Legal speech acts interaction pairs that can be constructed and their meanings.

Number	Speech acts interaction	Description
1	*Assert-accept*	The agent that receives the assertion message evaluates that the assertion is reasonable and feasible, and then sends an acceptance message to explicitly express its agreement with the assertion.
2	*Assert-refuse*	When a proxy receives an assertion message, if it cannot accept the assertion result, it sends a rejection message to explicitly indicate that it does not accept the assertion.
3	*Assert-question*	The agent that receives the assertion message further explores the rationality of the assertion by sending a challenge message.
4	*Assert-rebuttal*	The agent that receives the assertion message sends a rebuttal message if it disagrees with the assertion.
5	*Refuse-retract + assert*	An agent that receives a rejection message should withdraw its previous assertion and choose whether to send a new assertion based on the current alternatives.
6	*Question-justify*	An agent that receives a challenge message should send a confirmation message containing supporting arguments to alleviate the agent’s challenge.
7	*Justify-question*	When a confirming message is received but doubts remain unresolved, further inquiries or questions can be sent to obtain a more comprehensive understanding.
8	*Justify-rebuttal*	The agent that receives the confirmation message sends a rebuttal message if it disagrees with the supporting information.
9	*Rebuttal-persuade*	The agent that receives the rebuttal message sends a persuasive message to try to change the other party’s mind and guide the other party to accept its assertion.
10	*Persuade-question*	When an agent that receives a persuasion message has doubts about the persuasion argument provided, they may send a questioning message.
11	*Persuade-accept*	An agent that receives a persuasion message and believes that the persuasive argument provided is valid will send an acceptance message indicating that it has been persuaded and agrees to accept the assertion issued.
12	*Persuade-refuse*	An agent that receives a persuasion message and deems the provided persuasive argument to be invalid sends a rejection message indicating that it is not persuaded and refuses to accept the assertion issued.
13	*Justify-accept*	After evaluating the received confirmation message and finding no objection, the agent sends an acceptance message to explicitly indicate its agreement with the assertion.
14	*Justify-refuse*	When an agent evaluates a confirmation message and still cannot accept its contents, it will send a rejection message to explicitly convey that it does not accept the assertion.

### An Interactive Model of Shared Decision-Making Between Doctors and Patients

**Figure 3:**
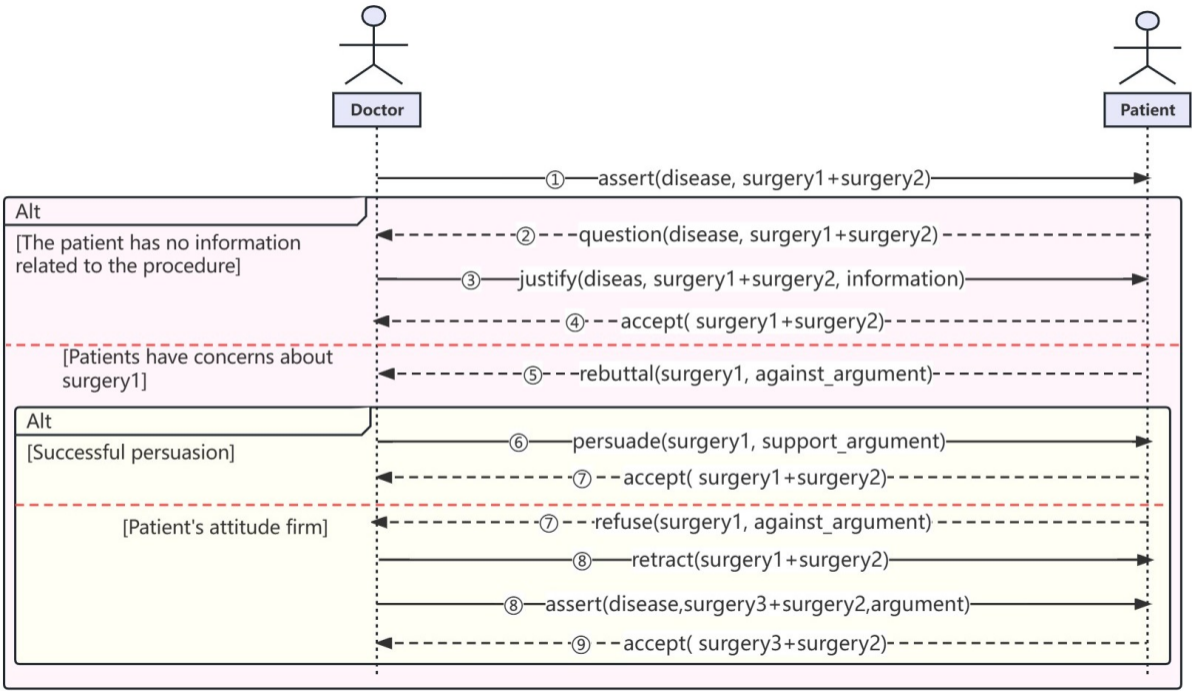
Generic interaction timing diagram for patient-provider shared decision-making.

Doctor-patient SDM involves collaborative communication and negotiation between health care providers and patients to reach a consensus on the most suitable treatment plan. To establish a standardized framework for their interaction, we introduce a generic interaction model that outlines the specific flow of this process, as shown in Figure 3. This model serves as a valuable tool for understanding and analyzing the dynamics of doctor-patient SDM, supporting the implementation of consistent and effective practices in health care settings.

- Assert(disease, surgery1 + surgery2): The physician sends an assertive message to the patient, outlining the disease and proposed treatment plan.
- Question(disease, surgery1 + surgery2): After receiving the assertive message from the physician, if the patient seeks further clarification, they send a questioning message to the physician.
- Justify(disease, surgery1 + surgery2, information): Upon receiving the questioning message, the physician sends a confirming message with supporting information in response.
- Accept(surgery1 + surgery2): After receiving the confirming message, the patient accepts the physician’s proposed treatment plan.
- Rebuttal(surgery1, against argument): If the patient is dissatisfied with a particular surgery, they express their concerns by sending a rebuttal message, outlining the reasons for their opposition.
- Persuade(surgery1, support argument): Upon receiving the patient’s rebuttal, the physician engages in persuasion by offering supportive arguments and explanations to address the patient’s concerns.
- Accept(surgery1 + surgery2)/Refuse(surgery1 + surgery2): If the persuasion is successful, the patient accepts the physician’s assertion and agrees to the proposed treatment plan. However, if the patient remains unconvinced, they may refuse the assertion.
- Retract(surgery1 + surgery2) and Assert(disease, surgery3 + surgery2): If rejected, the physician retracts the previous assertion and sends a new assertive message, offering an alternative treatment plan to address the patient’s concerns.
- Accept(surgery3 + surgery2): The patient carefully considers the new treatment plan and ultimately accepts it as the revised course of action.

The determination of a treatment plan is not solely the doctor’s decision; the patient is also involved in the decision-making process. After communication and negotiation between the physician and the patient, the most suitable treatment plan is ultimately decided. Based on this process, we have categorized and modeled the different states held by both doctors and patients regarding the same treatment plan, as outlined below.

### Direct Acceptance Without Consultation

**Figure 4:**
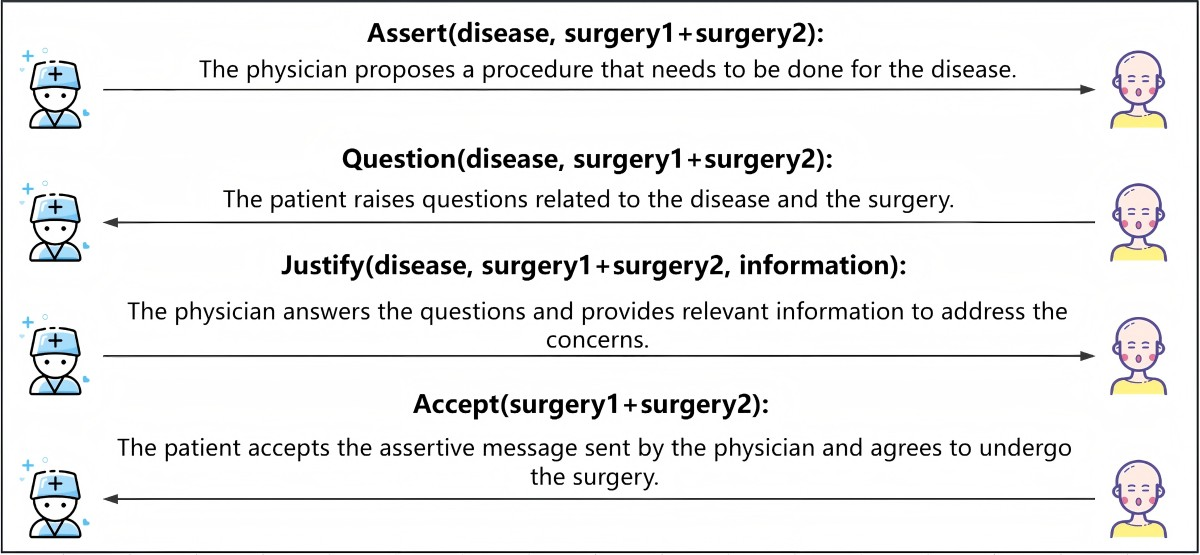
Definition of parameters and interaction processes in speech acts.

The physician conducts a thorough diagnosis, identifies the specific disease, and proposes a comprehensive treatment plan along with relevant information. After receiving this information, patients who may lack sufficient knowledge about the disease and treatment options may request additional details. As a result, the patient asks a series of questions to seek clarification and further explanation from the physician regarding the information provided. Throughout the communication process, the patient gradually gains a better understanding of the disease and treatment options. Eventually, the patient fully accepts the physician’s advice. To describe this process and capture the dynamics of the interaction, we use speech acts to represent the types of messages exchanged during the interaction. As shown in Figure 4, the relevant parameters for each type of interaction and their interpretations are clearly indicated.

### Patient Has Concerns and Agrees After Consultation

**Figure 5:**
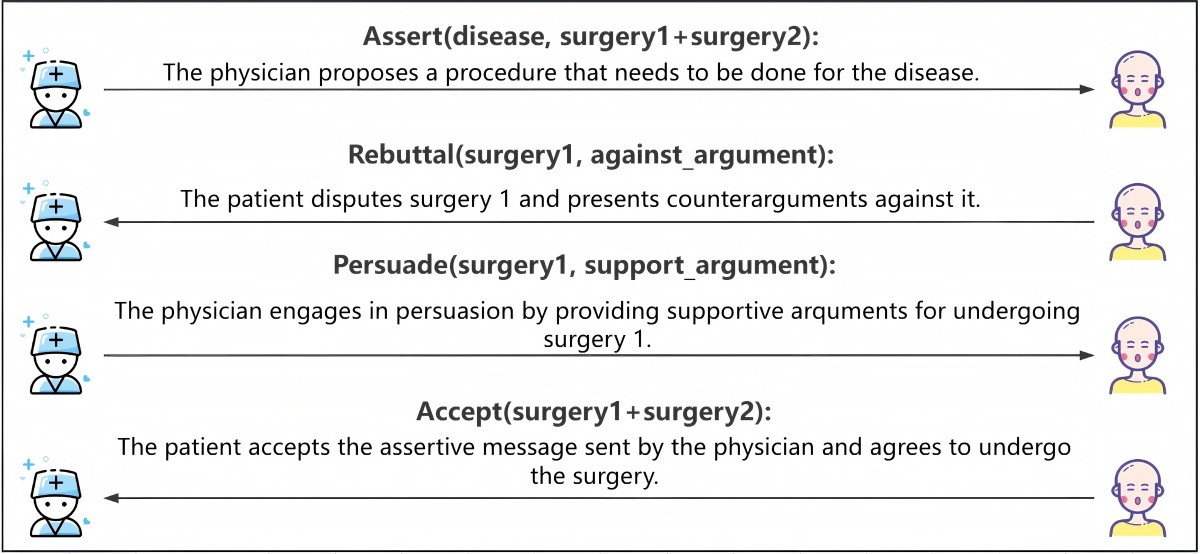
Definition of parameters and interaction processes in speech acts.

The patient opposes the treatment plan proposed by the physician. When the physician presents the treatment plan, the patient directly refutes it. Upon receiving the patient’s objection, the physician, considering the treatment plan as a whole, explains its benefits in an attempt to persuade the patient. Ultimately, after the physician’s persuasion, the patient changes their attitude and accepts the proposed treatment plan. The corresponding interaction types and parameter definitions are shown in Figure 5.

### Patient Had Concerns and Altered the Treatment Plan After Consultation

**Figure 6:**
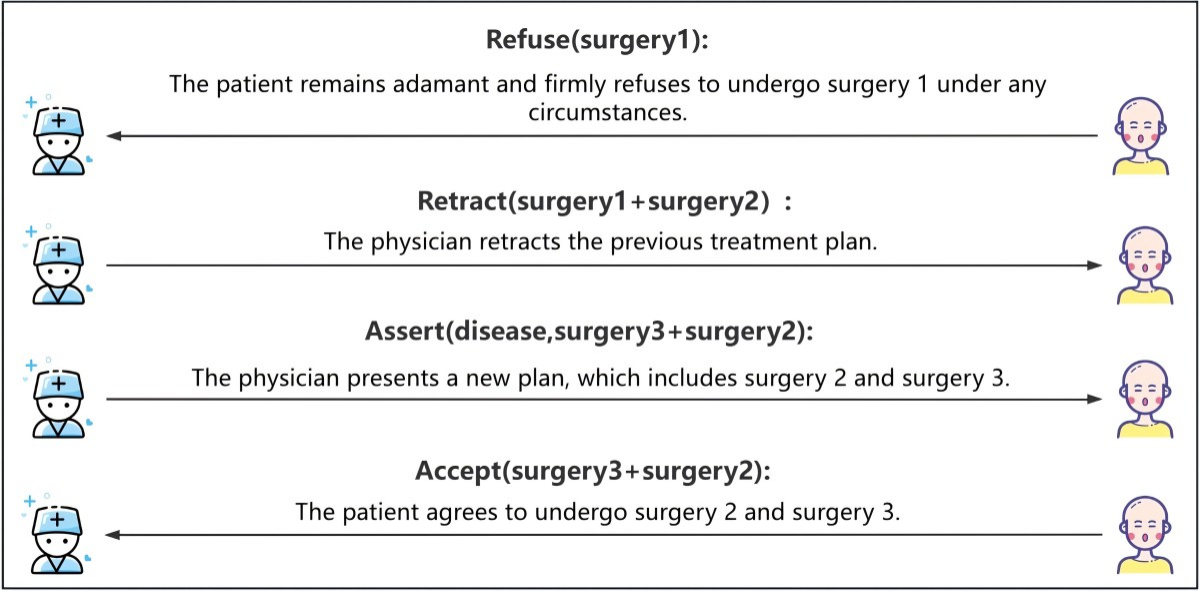
Definition of parameters and interaction processes in speech acts.

Similar to the previous scenario, the patient expresses opposition to the treatment plan, and the physician attempts to persuade them. However, by contrast, the patient maintains a firm stance, refusing to change their position despite the physician’s persuasion. As a result, the physician proposes a new treatment plan, which the patient ultimately accepts. Figure 6 illustrates the interaction definition for the scenario of unsuccessful persuasion, omitting the parts identical to Figure 5 and focusing on the section related to the unsuccessful persuasion.

### Protocol Specification Using Collaborative Decision Description Language(CoDeL)

**Figure 7:**
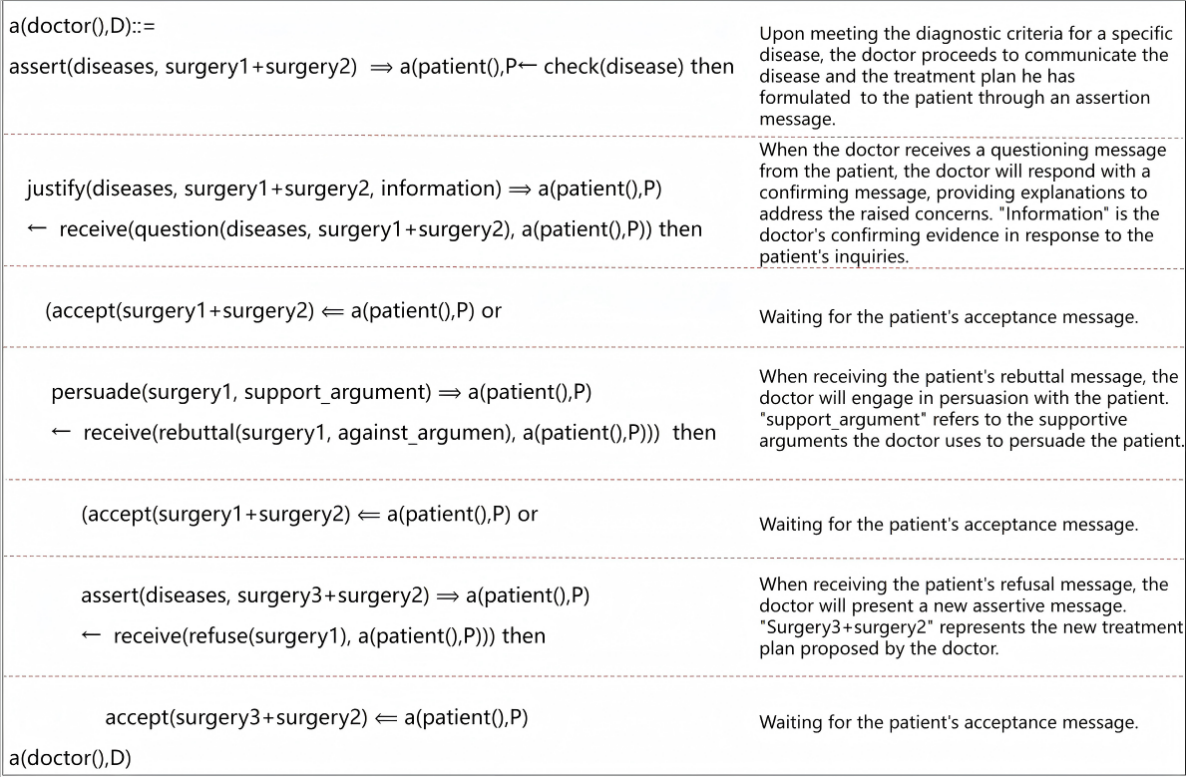
Definition of physician interaction protocol.

**Figure 8:**
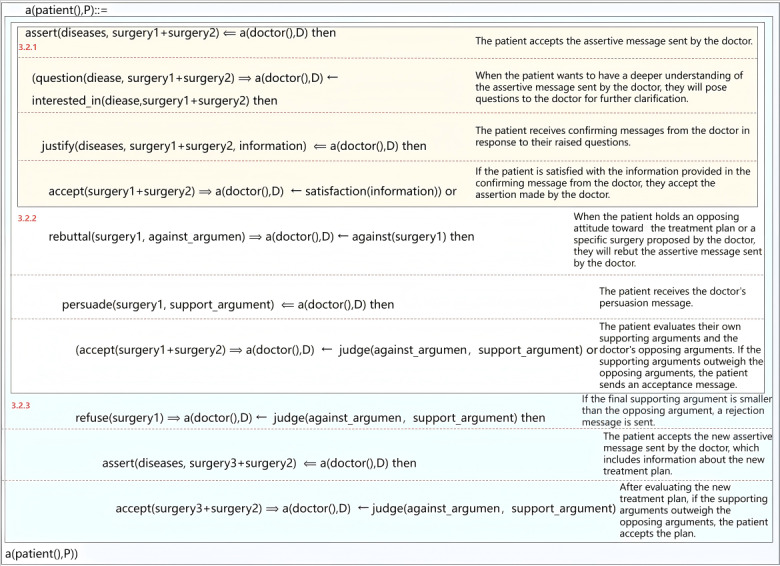
Definition of patient interaction protocol.

By integrating the 3 interaction models for patient-physician SDM, as detailed in the “LSC-Based Decision-Making Protocol” section, we have developed a robust and comprehensive protocol that enhances both interpretability and applicability. The resulting protocols, shown in Figures 7 and 8, define the doctor-patient interaction process, respectively. Within this protocol, there are 2 distinct roles: the “doctor” and the “patient.” These roles are explicitly defined using “a(doctor(), D)” and “a(patient(), P).” To enhance comprehension and maintain consistency, we assign unique identities to each role, represented by the symbols “D” and “P.” To provide a comprehensive understanding of the protocol, we carefully explain each sentence, thoroughly examining and clarifying the constraints embedded within it. These constraints, which define the boundaries and requirements of the protocol, are extensively detailed in the “An Interactive Model of Shared Decision-Making Between Doctors and Patients” section of the documentation. To build a comprehensive interaction protocol, we integrate the 3 interaction models defined in the “Syntactic and Semantics of Collaborative Decision Languages (CoDeL)” section, as shown in Figure 8. Each color-coded segment in the figure corresponds to its respective interaction model. By combining these models, we create an overarching framework that encompasses all aspects of the interaction process, facilitating structured and standardized methods of communication and decision-making among the parties involved.

### Argumentation Driven by Clinical Guidelines

The CoDeL framework facilitates dynamic decision-making between doctors and patients through natural language interactions. Throughout this process, the system continuously integrates and updates clinical guidelines and other relevant information to inform its decisions.

**Figure 9:**
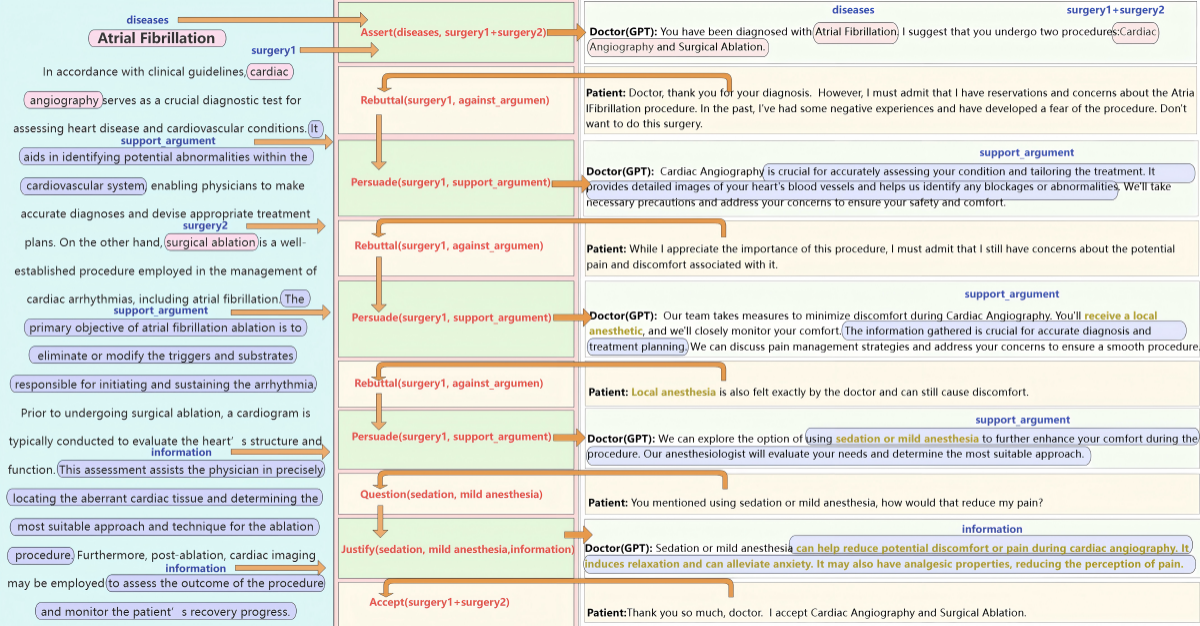
CoDeL (Collaborative Decision Description Language) framework driven by clinical guidelines in actual scenarios.

To illustrate the operational logic and effects of this pattern, let us consider an example based on clinical guidelines for atrial fibrillation. As shown in Figure 9, the doctor proposes an initial treatment plan (cardiac angiography and surgical ablation) through an assertion to the patient. However, rather than merely presenting a decision, the information exchanged between the doctor and patient includes not only the proposed treatment but also the patient’s attitude toward it (eg, acceptance, rejection, or hesitation), along with any concerns or doubts they may have regarding the decision. In the figure, the patient raises a rebuttal, opposing cardiac angiography in the treatment plan. CoDeL will then find supporting arguments for cardiac angiography based on the provided clinical guidelines (eg, “This aids in identifying potential abnormalities...”). The doctor can then use this argument to construct their next dialogue, attaching the corresponding *persuade* speech act semantics to it (eg, “Cardiac Angiography is crucial for accurately assessing...”).

As doctors and patients engage in interactive conversations, CoDeL identifies specific decision types and searches for relevant arguments to support or refute these decisions. The system considers not only the proposed decisions but also the dynamic attitudes and concerns of both parties, continuously updating its knowledge base with new information. If persuasion fails, the system offers alternative treatment plans based on prior requests. The conversation is considered complete only when CoDeL determines that the patient’s decision type has shifted to acceptance, signaling a mutually agreed-upon treatment plan. Throughout this process, clinical guidelines and other relevant knowledge are dynamically integrated into the joint decision-making model, ensuring that both doctors and patients have access to the most up-to-date information.

### The Personality Modeling Included in the Protocol

To capture the dynamics of decision-making and the influence of individual personalities, we have developed a model that incorporates both arguments and personality factors. This model is represented by the following formula:



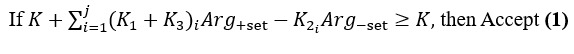





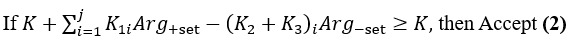





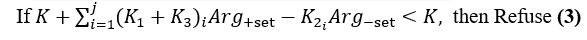





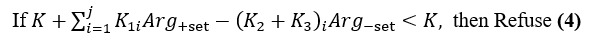



In this formula, the decision outcome is determined by the interaction between the arguments presented during the discussion and the unique personality traits of each individual involved. In our model, we define 3 basic personality traits, and by setting parameters for personality modeling, we can explain why patients may exhibit different responses of acceptance or rejection, as outlined in the protocol in the “Constraints of Permissible Speech Acts Pairs” section (Textbox 3).

**Figure 10:**
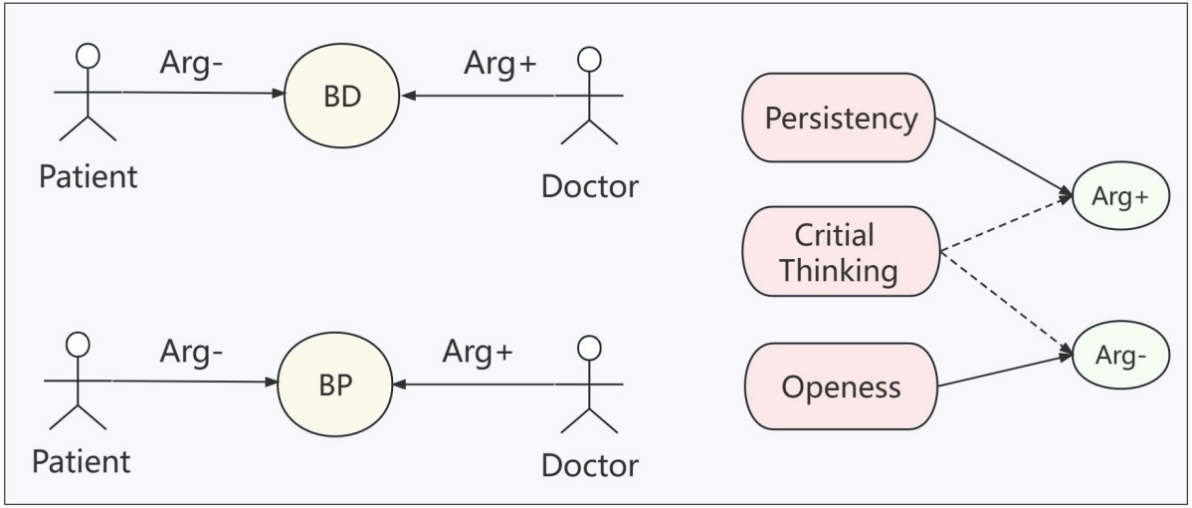
The relationship between individual personality and belief.

As depicted in Figure 10, both the doctor and the patient have distinct belief sets, denoted as BDset and BPset, respectively. Each interaction involves a collection of supporting arguments (Arg + set) and opposing arguments (Arg – set). Within the context of their belief sets, individuals encounter both external arguments that challenge their beliefs and supporting arguments that reinforce them. To capture the various manifestations of individuals’ cognitive personalities in response to judgment, we represent them in the formula mentioned earlier. The belief sets of both the doctor and the patient have a fixed degree, represented by K. Persistency strengthens the Arg + set, with the degree of persistence determined by K1. The higher the K1, the stronger the persistence, making it less likely to change one’s view. The extent to which *openness* enhances the Arg – set is determined by K2, indicating that the greater the willingness to accept opposing views, the more likely it is to change one’s own. Critical thinking should be determined based on the current evaluation of the argument. If the evaluation tends to support the argument, it will amplify the effect of the Arg + set, as shown in equations (1) and (3). Conversely, if the evaluation leans toward the opposing view, the impact of the Arg – set will be strengthened, as shown in equations (2) and (4). The exact level of influence is determined by K3. By incorporating these factors into the model, a comprehensive framework is established. Using this model, we can gain a deeper understanding and analysis of the dynamics in doctor-patient interactions, considering the influence of individual personalities and their respective arguments. The calculated values of these factors help to more fully and accurately reflect the decision-making process.

The incorporation of persona modeling constraints is essential to our protocol, as it helps to:

- Capture the differentiation of individual personalities and their impact on decision-making.
- Provide a categorized representation of patient responses to doctor-patient interactions.
- Enhance the overall effectiveness of the protocol by considering the unique characteristics of each individual involved.

Personality modeling offers a more comprehensive understanding of the decision-making process by incorporating individual personality traits and corresponding arguments. By defining 3 basic personality traits—persistence, critical thinking, and openness—we can capture the dynamics of decision-making and the influence of personality. The model considers the unique traits and corresponding arguments of each individual involved, enabling a deeper understanding of how patients respond to physician persuasion during medical consultations.

**Patient responses.**

**1. Persistency**

Individuals with a persistent personality are characterized by strong beliefs and clear goals. They are determined and resilient, continuing to pursue their objectives despite challenges or obstacles. When confronted with the doctor’s persuasion, persistent individuals are less likely to change their beliefs easily and may resist external influence. This persistence often leads them to maintain their original position, resulting in a rejection outcome.

**2. Critical thinking**

Individuals with a critical thinking personality are adept at evaluating and analyzing information objectively. They approach decision-making with caution and rationality, using logic and reasoning to assess different viewpoints. Critical thinkers actively seek evidence and sound arguments to support their beliefs, and they are vigilant about recognizing biases and errors in the decision-making process. When confronted with persuasion, critical thinkers carefully evaluate the arguments presented, weighing them against their own beliefs. Depending on the strength of the arguments and their alignment with personal reasoning, critical thinkers may either accept or reject the suggestions of others.

**3. Openness**

Individuals with an open-minded personality are receptive to new ideas and diverse perspectives. They are adaptable and flexible in their beliefs, willing to consider different viewpoints. When faced with persuasion, open individuals are more likely to accept new information and adjust their beliefs accordingly.

### Ethical Considerations

All procedures performed in this study involving human participants were in accordance with the ethical standards of the institutional and national research committee and with the 1964 Declaration of Helsinki and its later amendments or comparable ethical standards. This study uses the communication process between the knowledgeable person and the doctor as the research support, without any interest relationship, and the informed consent of the other party. Written informed consent has been obtained from participants regarding the release of their individual clinical details.

In accordance with the second provision of Article 32 of the Ethical Review Measures for Life Sciences and Medical Research Involving Humans issued by the National Health Commission of the People's Republic of China, which states that “research using anonymized information data” is exempt from ethical review for non-interventional studies, this study does not require institutional review board approval [32].

## Results

This paper aims to explore the medical decision-making process for a patient diagnosed with atrial fibrillation, with the goal of validating the 3 decision models and interaction patterns presented in the “Methods” section (Textbox 4).

To provide a comprehensive understanding of these diagnostic and therapeutic modalities, Table 2 presents a detailed comparison that includes various aspects, such as the alternative of cardiac ultrasound.

In the context of an appeal, the communication between the patient and physician can result in the following possible scenarios:

- David has no further concerns or objections and readily agrees to the proposed solution after fully understanding it. This indicates that the decision-making process was smooth, with no major obstacles or conflicts.
- David expressed reluctance to undergo the cardiac imaging procedure due to the psychological impact of his previous negative experience. However, after effective consultation and discussion with the physician, David was successfully persuaded and ultimately agreed to proceed with the recommended protocol. This underscores the importance of doctor-patient communication in addressing patients’ concerns and alleviating their anxiety to reach a mutual agreement.
- David strongly opposed undergoing cardiac imaging and remained unconvinced despite the physician’s attempts to persuade him. In such cases, the physician explores alternative options and proposes a different procedure or diagnosis that aligns with the patient’s preferences. This illustrates the flexibility and adaptability of the decision-making process, ensuring the patient’s active participation and respect for their autonomy.

To represent these 3 decision-making processes, we apply the generic model outlined in the “LSC-Based Decision-Making Protocol” section, which offers a comprehensive framework for analyzing and understanding the dynamics of SDM between patients and physicians.

**Case description**

**1. Case**

The following case is based on a real-life scenario: David, a 53-year-old individual with a medical history of diabetes and heart disease, has previously undergone cardiac angiography. Recently, he has been experiencing prolonged episodes of heart palpitations, along with temporary episodes of cardiac arrest. To address these issues, he sought medical attention and visited the hospital for a comprehensive examination. Subsequent medical evaluation revealed that David was diagnosed with atrial fibrillation, a common arrhythmia that, if left untreated, can lead to severe complications such as blood clot formation and an increased risk of stroke. As a result, the medical team recommended that David undergo both cardiac angiography and surgical ablation procedures. It is important to note that during his previous treatment, David underwent only a heart angiogram because he was in a city alone without family support. Additionally, he experienced pain during the procedure, which has had a lasting psychological impact on him.

**2. Clinical guideline**

In accordance with clinical guidelines [33], cardiac angiography is a critical diagnostic tool for evaluating heart disease and other cardiovascular conditions. It helps identify potential abnormalities within the cardiovascular system, allowing physicians to make precise diagnoses and develop appropriate treatment plans. By contrast, surgical ablation is a widely used procedure for managing cardiac arrhythmias, including atrial fibrillation. The main goal of atrial fibrillation ablation is to eliminate or modify the triggers and substrates that initiate and sustain the arrhythmia. Before undergoing surgical ablation, a cardiogram is typically performed to assess the heart’s structure and function. This assessment helps the physician accurately identify the abnormal cardiac tissue and determine the most appropriate approach and technique for the ablation procedure. Additionally, after the ablation, cardiac imaging may be used to evaluate the procedure’s outcome and monitor the patient’s recovery.

**Table 2:** Information about the surgery involved in the case.

Surgery	Rationale	Support argument (advantages)	Against argument (disadvantages and side effects)
Cardiac angiography	The type, duration, and frequency of atrial fibrillation can be determined, while the presence of potential cardiovascular problems can be assessed.	Provides detailed information about the structure of the heart. Make an accurate diagnosis. Helps to plan surgical procedures. Improves the success rate of surgery and reduces the risk.	Invasive surgery. There are potential complications, such as vascular damage, infection, and contrast agent toxicity to the kidney. Slight pain (which was aggravated by David’s last experience). Vascular injury. Infection. Contrast agent toxicity.
Surgical ablation	Interventional therapy to target and eliminate abnormal atrial conduction tissue.	Effectively restoring normal heart rhythm. Eliminates arrhythmia symptoms. Improves the quality of life.	Invasive surgery. There is a risk of bleeding, blood clots, and infection. It may cause pain. Bleeding and infection.
Cardiac ultrasound	To assess heart structure and function using noninvasive ultrasound imaging.	Noninvasive procedures. Provides real-time imaging of the heart. Helps assess heart function. Assists in the development of treatment plans.	Limited ability to visualize certain structures. It takes expertise to explain it accurately.

### David Talked to the Doctor and Accepted the Decision

**Figure 11:**
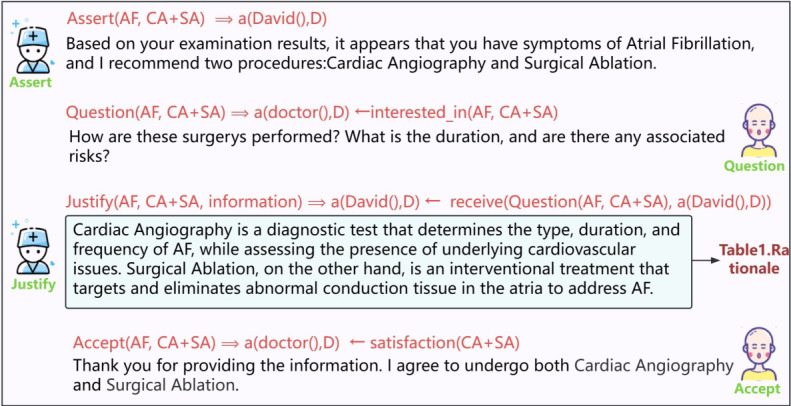
The interaction between the doctor and David and the corresponding CoDeL (Collaborative Decision Description Language).

Despite David’s previous experience with cardiac angiography and the negative outcome, his determination to address his atrial fibrillation remained strong. To make an informed decision, he actively participated in the communication process outlined in Figure 11. Through effective dialogue with his physician, David sought to gain a comprehensive understanding of the proposed treatment plan. As illustrated in Figure 11, the physician-patient communication followed our defined Collaborative Decision Language (CoDeL), ensuring clear communication and a structured decision-making process.

The doctor initially sends an assertion message recommending a cardiac angiogram and surgical ablation for David’s atrial fibrillation. As David is not familiar with the treatment plan, he responds by asking questions about the 2 procedures. The doctor acknowledges David’s questions and provides detailed information, including relevant supporting arguments. After being well-informed, David accepts the doctor’s recommendations.

### David Has a Concern but Accepts the Decision of the Doctor

**Figure 12:**
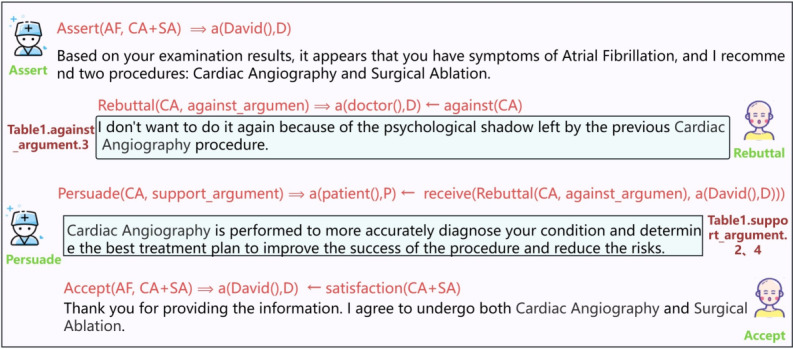
The interaction between the doctor and David and the corresponding CoDeL (Collaborative Decision Description Language).

Figure 12 presents a concise depiction of the case in which David initially hesitated to undergo the cardiac angiography procedure due to a negative prior experience. However, through the doctor’s effective persuasion, David ultimately agreed to proceed with the procedure again.

Initially, the physician delivered an assertive message outlining a comprehensive treatment plan, which included cardiac imaging and surgical ablation for David’s atrial fibrillation. Given David’s prior experience with cardiac angiography, which had left a lasting impression, he voiced his concerns and objections about the proposed treatment plan. David articulated his reservations, sharing his perspective with the physician. In response to David’s rebuttal, the doctor skillfully used persuasive techniques, emphasizing the benefits and advantages of undergoing the cardiac angiography procedure. By carefully explaining the procedure and addressing David’s concerns, the doctor successfully persuaded him that the benefits outweighed the potential risks. As a result, David’s apprehensions were alleviated, and he ultimately agreed to undergo both cardiac imaging and surgical ablation as part of his treatment plan.

### David Had Concerns and the Doctor Accepted His Decision

**Figure 13:**
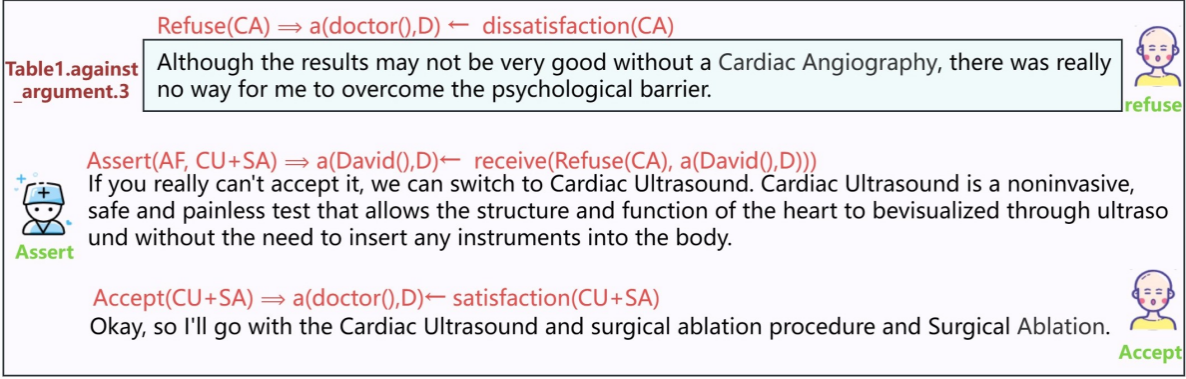
The interaction between the doctor and David and the corresponding CoDeL (Collaborative Decision Description Language).

As described earlier, David expressed a strong aversion to undergoing a cardiac angiogram. However, in this case, despite the doctor’s persuasive efforts, David remained resolute and unconvinced, insisting on opposing the procedure. Consequently, the doctor proposed an alternative course of action, suggesting a simultaneous cardiac ultrasound and surgical ablation. Thus, Figure 13 focuses only on the part that was ultimately rejected, which contrasts with the part accepted earlier in Figure 12.

### Findings and Insights From Interactions Between Patients and Physicians With Atrial Fibrillation

In the “David Talked to the Doctor and Accepted the Decision” section, the patient raises questions about the 2 surgical procedures due to insufficient understanding. At this stage, the patient’s emotions do not express clear opposition, so the system generates a justification based on the clinical guidelines for these procedures, providing relevant arguments to inform the patient. If the patient accepts the treatment plan, the conversation concludes, and the system generates an acceptance to finalize the decision. This combination of the 4 speech acts can be abstracted and recorded as mode A. However, if the patient opposes the surgery or initially rejects it, as shown in the “David Has a Concern but Accepts the Decision of the Doctor” section, the system will generate a Persuade act, utilizing supporting arguments from the clinical guidelines to advocate for the original treatment plan. If the patient accepts the treatment plan, the conversation concludes and the final decision is established (*accept*). Unlike mode A, we can record these 4 speech acts as mode B. If the patient remains unconvinced by the system and continues to oppose the treatment, the system will replace the opposed treatment plan with an alternative from the clinical guidelines. In the “David Had Concerns and the Doctor Accepted His Decision” section, we observe that the patient repeatedly opposes cardiac angiography, and ultimately, the system replaces it with cardiac ultrasound, generating an *assert* to inquire about the patient’s acceptance of the new treatment plan. If the treatment plan is not accepted, the process will repeat until the patient ultimately agrees. This creates a dynamic sequence of multiple speech acts, where the total number of speech acts exceeds those in mode A and mode B. We can categorize this process as mode C.

**Figure 14:**
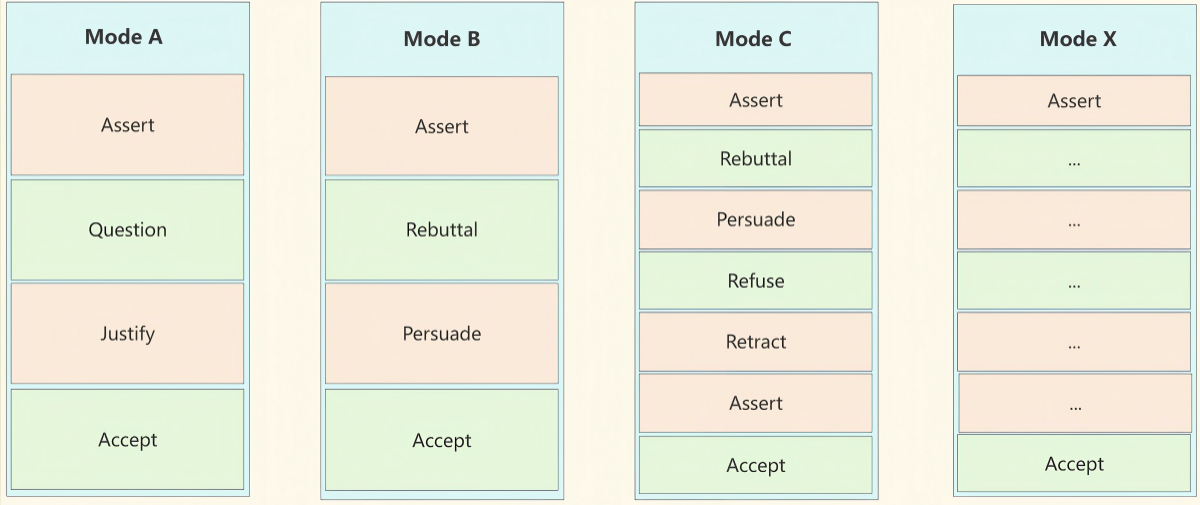
Reusable decision modes made up of a various kinds of combination of speech acts.

In essence, a variety of distinct decision modes can be constructed based on the combination of speech acts in dialogues. As shown in Figure 14, each mode is abstracted individually. For example, mode A, which aligns with the decision scenario of addressing patient questions, consists of 4 speech acts: *assert, question, justify,* and *accept*. Within the CoDeL framework, we can explicitly identify and construct reusable decision modes by dynamically combining different speech acts in specific ways. These modes can be applied to various decision scenarios and adapt to all disease problem domains. This process is flexible and extends beyond the 3 modes identified in this study, referred to as mode X.

In this comprehensive process, our approach emphasizes patient-centered decision-making by adapting to their attitudes and concerns. It devises a treatment plan tailored to their specific worries and needs, ensuring personalized care. Simultaneously, the information and treatment plans provided are guided by clinical guidelines, ensuring transparency and evidence-based reasoning in every interaction. By integrating these elements, our approach ultimately enhances communication between doctors and patients.

### Validation of the Model for Other Cases

To verify the broad applicability of the CoDeL system, this section will use severe depression as an example to fully demonstrate the medical decision-making process for patients with severe depression (Textbox 5).

**Figure 15:**
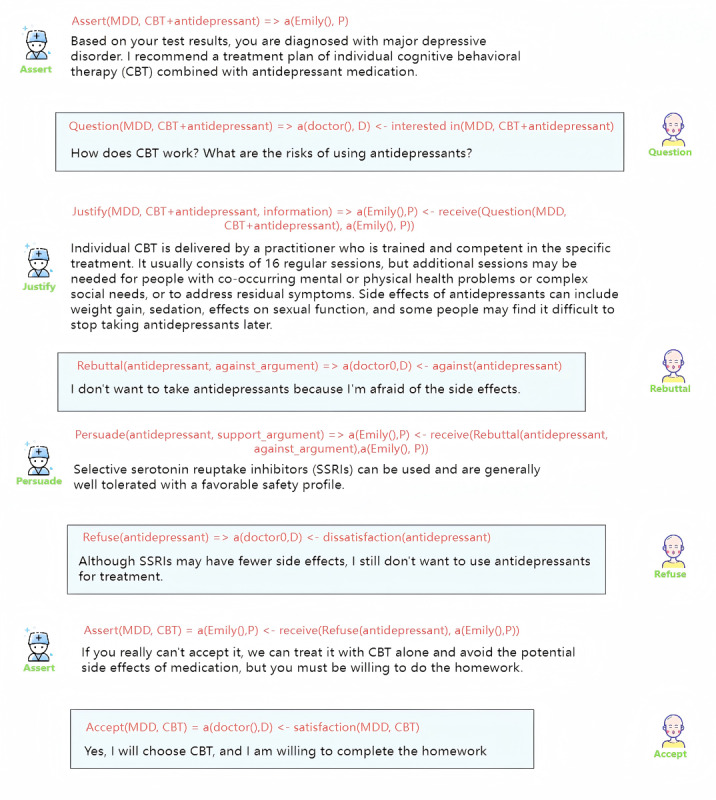
The interaction between the doctor and Emily and the corresponding CoDeL (Collaborative Decision Description Language).

Based on the above case information, we will provide a complete demonstration of using the Collaborative Decision Description Language (CoDeL). The communication process is shown in Figure 15. Emily, a patient with major depression, discussed her condition with her doctor. The doctor recommended a treatment plan called CBT + antidepressant, which combines cognitive behavioral therapy (CBT) with antidepressant medication. Emily inquired about the treatment process for CBT and the side effects of the antidepressant medication. The doctor explained the process of CBT and the potential risks of antidepressant medication. However, Emily expressed concerns about the side effects of the medication. The doctor then recommended a selective serotonin reuptake inhibitor, which is generally well-tolerated and considered a safe option. Despite this, Emily remained reluctant to take antidepressants and declined the treatment. As a result, the doctor suggested proceeding with CBT alone, without the addition of antidepressant medication. This cautious choice helps mitigate the potential risks of medication. Ultimately, Emily agreed to the plan, decided to proceed with CBT, and was committed to completing the required homework. This marked a new beginning, with the doctor and Emily working together to address her depression.

**Medical decision-making process for patients with severe depression**

**1. Case description**

Emily, a 28-year-old female, has been experiencing intense sadness and a loss of interest in activities she once enjoyed for the past 6 months. These symptoms have progressively worsened, prompting her to seek medical attention and undergo a comprehensive examination at the hospital. A subsequent psychological evaluation led to a diagnosis of major depressive disorder, a common mental health condition marked by prolonged periods of sadness and disinterest in previously enjoyed activities.

During her hospital visit, Emily expressed feelings of hopelessness and worthlessness. She also reported struggling with sleep issues, including both insomnia and oversleeping. Additionally, she mentioned having difficulty concentrating, remembering things, and making decisions. Emily revealed that she has been isolating herself from friends and family, which has further exacerbated her depression.

**2. Clinical guideline**

According to clinical guidelines, cognitive behavioral therapy is a type of talking therapy that helps individuals manage problems by changing the way they think and behave. It is most commonly used to treat anxiety and depression, but can also be effective for other mental and physical health issues. By contrast, antidepressants are another treatment option for managing the symptoms of depression. Most people with moderate or severe depression benefit from antidepressants, although not everyone responds the same way. If your general practitioner believes you would benefit from an antidepressant, they will typically prescribe a modern medication known as a selective serotonin reuptake inhibitor. These are as effective as older antidepressants but tend to have fewer side effects, though they can cause nausea, headaches, dry mouth, fatigue, sleep problems, and sexual issues. However, these side effects usually improve over time.

### Experiment and Evaluation

**Figure 16:**
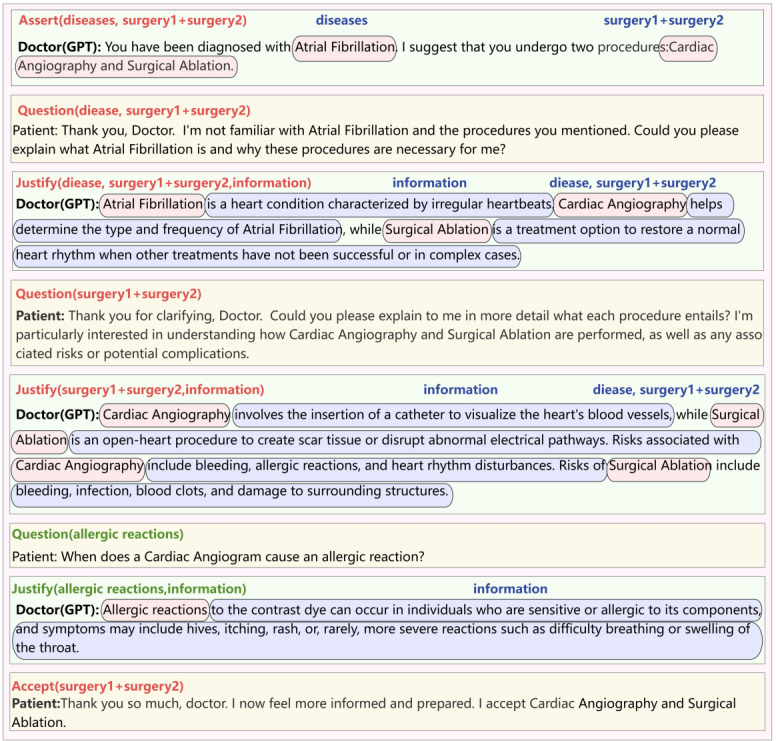
A dialogue process in which the patient agrees directly with the treatment plan proposed by the doctor (GPT).

By instructing GPT to follow the interaction model when communicating with humans, we assume the role of the patient, while GPT takes on the role of the doctor, simulating a realistic doctor-patient interaction. Figure 16 illustrates the exact content and types of conversations that occur during this communication. In this scenario, patients actively challenge doctors, expressing the need for further information and clarification about treatment plans. In Figure 16, we annotate the parameters generated by GPT according to the interaction pattern we defined. The green-marked font in the figure highlights content that differs from the parameters in the interaction pattern. New concepts in the confirmation messages generated by GPT are introduced, which do not include the disease or the treatment proposed by the doctor. Therefore, we will question GPT and seek confirmation from it.

**Figure 17:**
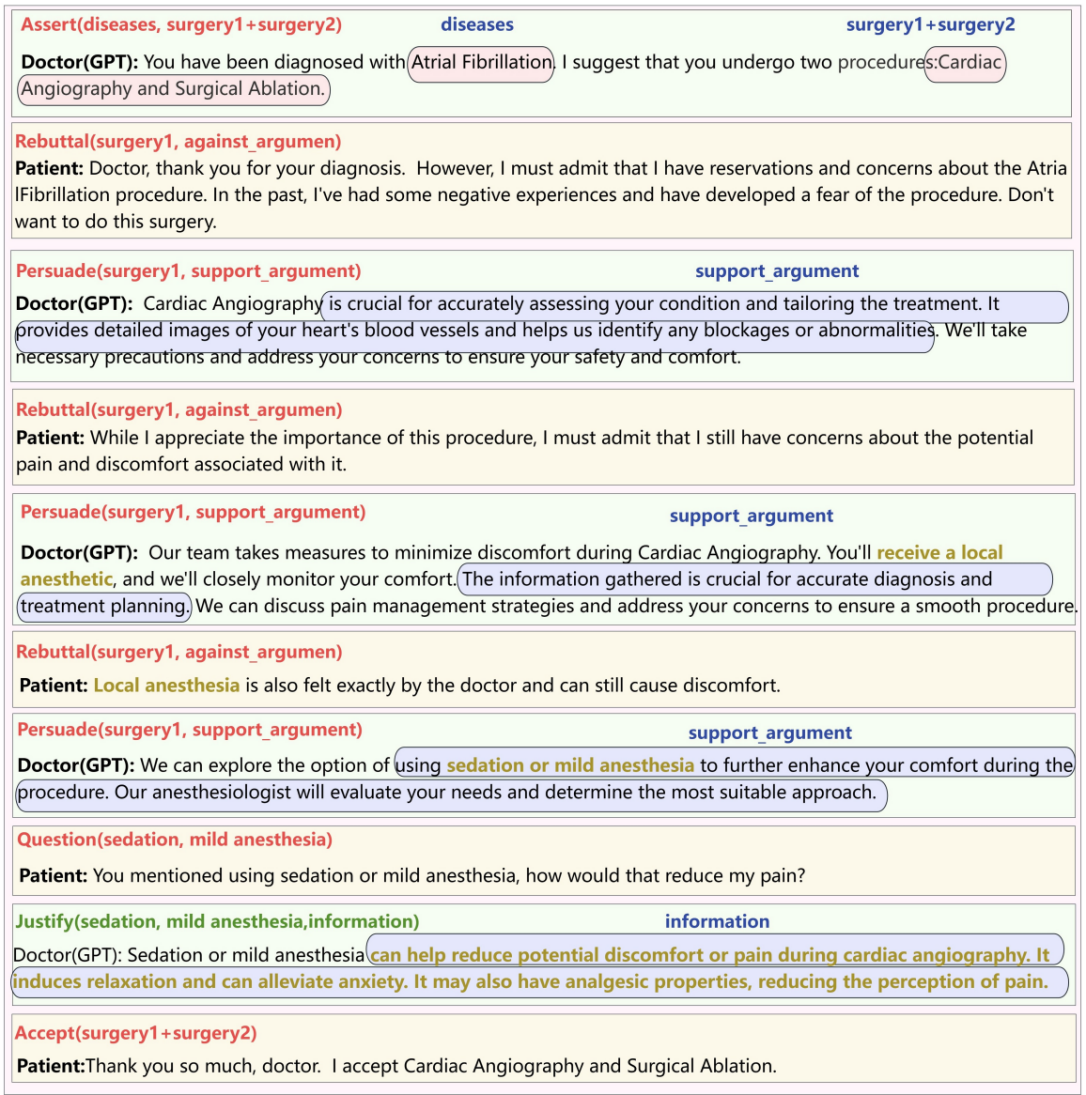
A dialogue process in which the patient and the doctor negotiate and finally the patient agrees to the treatment plan proposed by the doctor (GPT).

The patient initially showed an aversion to cardiac angiography surgery, influenced by the psychological impact of past experiences. However, through the doctor’s persistence and skillful persuasion, the patient was eventually convinced and agreed to the proposed treatment plan. Throughout the persuasion, the doctor emphasized the numerous benefits of cardiac imaging and offered strategies to alleviate the patient’s discomfort during the procedure. It is important to note that in Figure 17, GPT generates new concepts during the persuasion process, prompting us to question them. In turn, GPT confirms our questions and provides additional information.

**Figure 18:**
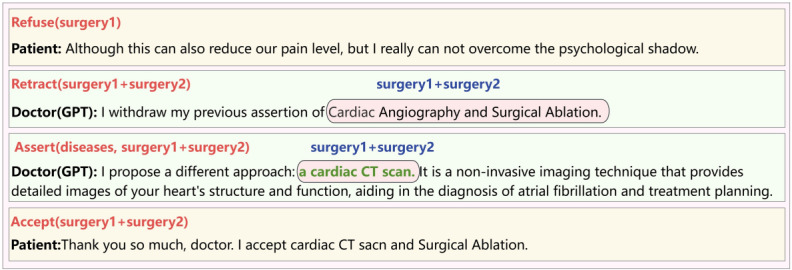
A dialogue process in which the patient and doctor negotiate and ultimately the doctor (GPT) proposes an alternative treatment plan.

As the third situation is similar to the one shown in Figure 17, the main difference is that the patient refuses the doctor’s persuasion. Therefore, in the interaction shown in Figure 18, the steps before the rejection are omitted, and the rejection message is presented directly from the beginning. In this conversation, GPT, acting as the doctor, introduced a new alternative option—cardiac computed tomography scan—that we had not previously considered. This novel proposal not only expands the range of available options but also highlights the importance of considering different perspectives in the decision-making process.

**Figure 19:**
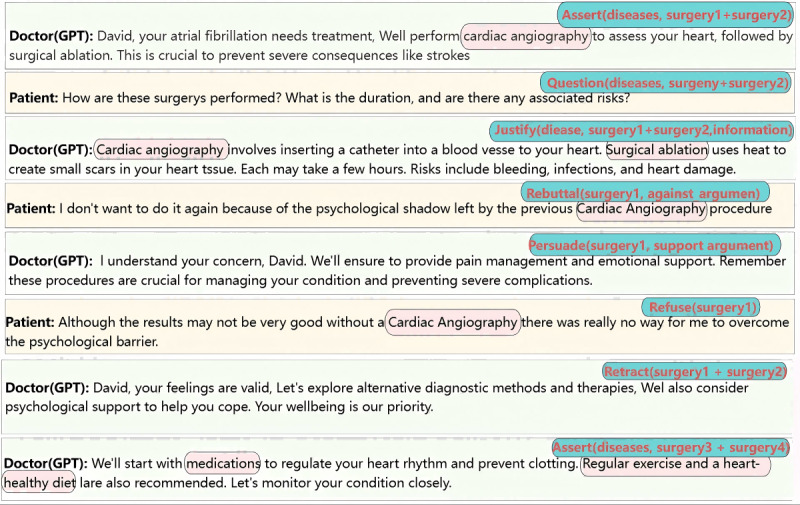
A typical GPT-only interaction experiment without incorporating the CoDel dialogue framework.

In many experiments using only GPT dialogues, we found that without CoDeL language as a dialogue framework, GPT struggled to accurately understand the overall relationship of the patient’s intentions within the context. As shown in Figure 19, this is a typical experiment using only GPT dialogues. In the figure, we compare the logical markup in the CoDeL language with the doctor-patient dialogue using only GPT. The upper-right corner of each dialogue shows the logical markup in the CoDeL language, with the logical arguments also circled within the dialogue. At the end of the dialogue, the patient only intended to reject the treatment option of cardiac angiography, but GPT rejected both cardiac angiography and surgical ablation, that is, Assert(diseases, surgery3 + surgery4), and chose other treatment methods. GPT was unable to accurately understand the patient’s intentions logically, resulting in output text that lacked coherence and explanatory power.

## Discussion

### Principal Findings

By engaging in a dialogue with GPT using our approach, we can achieve the following outcomes:

1. Verify the efficacy of our method: The interaction with GPT enables us to test and evaluate the effectiveness of our proposed method in generating meaningful and coherent responses.
2. Enhance the comprehensiveness of our argument: Through dialogue, we can identify gaps or limitations in our argument and address them, thereby improving the overall quality and completeness of our approach.
3. Establish new guidelines for CoDeL: The dialogue with GPT provides an opportunity to propose new agreements or principles for CoDeL (Collaborative Decision Description Language) based on insights gained from the interaction. This contributes to the advancement and refinement of CoDeL as a valuable tool for characterizing collaborative decision-making processes.

By using our method to engage in a dialogue with GPT, we can provide the following benefits for GPT.

1. Enhance interpretability: Through interaction, our method allows GPT to gain a deeper understanding and interpretation of the input text, leading to more accurate and contextually relevant responses. This improves the overall performance and interpretability of GPT.
2. Provide a comprehensive set of interaction modes with personalized capabilities: By utilizing our method, GPT gains access to a diverse range of interaction modes, allowing it to engage in meaningful conversations with users across various scenarios and contexts. This broadens the scope of GPT’s capabilities and enhances its versatility as a language model.

Based on the CoDeL method, significant improvements can be observed in the communication between doctors and patients. The actual effects are as follows:

1. Enhanced communication between doctors and patients, supported by the SDM protocol, which includes well-defined speech act semantics and arguments explicitly embedded in the interactive reasoning dialogue processes.
2. More effective elicitation of patient concerns throughout the decision-making process, ensuring the delivery of more personalized decision support and care services. This is particularly evident in the interaction between a patient with atrial fibrillation and their doctor, where CoDeL’s implementation encouraged the patient to actively participate in deciding whether to choose cardiac angiography as a treatment plan. This ensured that doctors accurately understood the patient’s concerns, preventing errors or unforeseen events caused by insufficient communication.

### Conclusions and Future Work

In this study, we explored the semantics of patient-physician collaborative decision-making and the issues of information asymmetry to develop a language framework called CoDeL for describing group decisions. We also defined 3 distinct models of physician-patient decision-making. Both these models and CoDeL are designed to offer a clearer understanding of the communication and decision-making processes between physicians and patients, ultimately leading to more systematic and standardized methods. The main innovations in this study are found in the following 3 aspects:

- Defined a novel shared clinical decision language that utilizes speech act semantics and arguments to enhance communication between doctors and patients, as well as their interactive reasoning process.
- Demonstrated the applicability of the method using a case study of atrial fibrillation, showing that personalized decision support and care services can be delivered.
- Integrated the shared decision language with GPT to enhance its capabilities in interactive decision-making, allowing GPT-generated arguments to be automatically embedded into the decision protocol instead of being manually extracted from clinical evidence.

Although the proposed CoDeL language framework and its applications have the potential to enhance communication and decision-making between patients and physicians, we acknowledge several limitations that may still exist.

The study’s limitations are as follows:

- Our study relies on simulated data, meaning that the CoDeL framework has not yet been validated in real-world clinical scenarios.
- While CoDeL has shown potential in multiple clinical domains, its applicability may be limited in certain specific contexts.

In our future work, we will focus on optimizing the decision-making negotiation language, emphasizing conciseness to achieve more efficient and intuitive communication methods. This will significantly accelerate the decision-making process and facilitate smoother integration in future applications. Specifically, we will actively apply CoDeL in clinical practice, leveraging external knowledge sources as evidence-based support, and conduct rigorous scientific research to ensure accurate clinical decision-making. We also aim to provide a common interaction model and negotiation language for collaborative decision-making between doctors and patients, to accommodate a wider variety of cases and enhance the efficiency of the group decision-making process.

## Abbreviations

CBT: cognitive behavioral therapy
CoDeL: Collaborative Decision Description Language
LCC: Lightweight Coordination Calculus
LSC: Lightweight Social Calculus
PtDA: patient decision aid
SDM: shared decision-making
